# CCK and GLP-1 release in response to proteinogenic amino acids using a small intestine ex vivo model in pigs

**DOI:** 10.1093/jas/skac093

**Published:** 2022-03-22

**Authors:** Maximiliano Müller, Elout Van Liefferinge, Marta Navarro, Elisabet Garcia-Puig, Alan Tilbrook, Robert van Barneveld, Eugeni Roura

**Affiliations:** 1 Centre of Nutrition & and Food Sciences, Queensland Alliance for Agriculture and Food Innovation (QAAFI), The University of Queensland, Brisbane, QLD 4072, Australia; 2 Laboratory of Animal Nutrition and Animal Product Quality (LANUPRO), Department of Animal Sciences and Aquatic Ecology, Ghent University, Ghent, Flanders 339000, Belgium; 3 Centre for Animal Science, Queensland Alliance for Agriculture and Food Innovation (QAAFI) and the School of Veterinary Science, The University of Queensland, Brisbane, QLD 4072, Australia; 4 SunPork Group, Brisbane, QLD 4009, Australia

**Keywords:** amino acid, cholecystokinin, gastrointestinal tract, glucagon-like peptide 1, pig

## Abstract

The impact of individual amino acids (**AA**) on gut hormone secretion and appetite regulation in pigs remains largely unknown. The aim of the present study was to determine the effect of the 20 proteinogenic AA on the release of the anorexigenic hormones cholecystokinin (**CCK**) and glucagon-like peptide 1 (**GLP-1**) in postweaning pigs. Six 25-d-old male piglets (Domestic Landrace × Large White; body weight = 6.94 ± 0.29 kg) were humanely killed for the collection of intestinal segments from the duodenum, jejunum, and ileum. Tissue samples from the three intestinal segments were used to determine which of the regions were more relevant for the analysis of gut peptides. Only the segments with the highest CCK and GLP-1 secretion and expression levels were evaluated with the 20 individual AA. Tissue segments were cut open, cleaned, and stripped of their muscle layer before identical circular samples were collected and incubated in 24-well plates for 1 h (37 °C, 5% v/v CO_2_). The culture broth consisted of a glucose-free KRB buffer containing no added AA (control) or with the addition of 10 mM of 1 of the 20 proteinogenic AA. Following incubation, tissues and supernatant were collected for gene expression and secretion analysis of CCK and GLP-1 levels. CCK secretion and mRNA expression were higher (*P* < 0.05) in duodenum when compared with proximal jejunum or ileum, whereas GLP-1/proglucagon levels were higher in ileum vs. duodenum (*P* < 0.05) and jejunum (*P* < 0.05, for GLP-1 only) in postweaning pigs. Based on these results, the effect of AA on CCK and GLP-1 secretion was studied in the duodenum and ileum, respectively. None of the AA tested stimulated both anorexigenic hormones. Of all the essential AA, Ile, Leu, Met, and Trp significantly (*P <* 0.05) stimulated GLP-1 from the ileum, while only Phe stimulated CCK from the duodenum. Of the nonessential AA, amide AA (Gln and Asn) caused the release of CCK, while Glu and Arg increased the release of GLP-1 from the ileum. Interpreting the results in the context of the digestion and absorption dynamics, non-bound AA are quickly absorbed and have their effect on gut peptide secretion limited to the proximal small intestine (i.e., duodenum), thus, mainly CCK. In contrast, protein-bound AA would only stimulate CCK release from the duodenum through feedback mechanisms (such as through GLP-1 secreted mainly in the ileum).

## Teaser

The individual impact of the 20 proteinogenic amino acids (**AA**) on the secretion of the anorexigenic gut hormones cholecystokinin and glucagon-like peptide 1 is described in postweaning pigs using a small intestine ex vivo model. Both non-bound essential and nonessential AA stimulated gut hormone release, demonstrating they play a key role in appetite control in pigs.

## Introduction

The postweaning process has been associated with a high degree of stress and poor performance with potential lifetime consequences in pigs (Collins et al., 2017). There are multiple factors involved, including low early feed intake after weaning followed by overconsumption, impaired intestinal function, and diarrhea (Bruininx et al., 2001; King and Pluske, 2003). In addition, piglet diets are often relatively rich in crude protein and/or synthetic amino acids (**AA**). Some dietary AA have been shown to significantly impact pig’s appetite (Roura et al., 2011). Furthermore, the gastrointestinal sensing of AA and peptides derived from protein digestion can stimulate the secretion of gut hormones involved in appetite regulation (Müller et al., 2021). However, little is known about how individual AA affect gut peptide secretion in pigs (Roura et al., 2016). Consequently, studying the anorexigenic properties of AA and the physiological mechanisms involved have the potential to help uncover the control of feed intake in postweaning pigs.

Cholecystokinin (**CCK**) and glucagon-like peptide 1 (**GLP-1**) are two of the better-known anorexigenic hormones produced by the gastrointestinal tract (**GIT**) in pigs and other mammalian species. CCK and GLP-1 are primarily secreted by enteroendocrine cells (**EEC**) located in proximal or distal segments of the small intestine, respectively. CCK is mainly released by the duodenum (I-cells), while GLP-1 is released by the ileum and colon (L-cells) (Bacarese-Hamilton et al., 1984; Voortman et al., 2012; Pluschke et al., 2018). In mice, Phe, Trp, Leu, and Glu have previously been shown to stimulate CCK release by stimulating the calcium-sensing receptor (**CaSR**) or the umami taste receptor (T1R1-T1R3; Wang et al., 2011; Daly et al., 2013). Similarly, in vitro evidence in rodents and humans suggests that a considerable group of AA trigger GLP-1 secretion including, Trp, Phe, Asn, Gln, Glu, Ala, Ser, Leu, Ile, Met, and Arg (Reimann et al., 2004; Chen and Reimer, 2009; Mace et al., 2012). In pigs, Arg stimulated duodenal CCK release via activation of CaSR (Wang et al., 2018). Similarly, Phe and Trp increased the mRNA expression of CaSR and the release of CCK in the duodenum, whereas Leu and Ile induced CCK release through the activation of T1R1-T1R3 in the jejunum (Zhao et al., 2018; Feng et al., 2019; Tian et al., 2019). In contrast, little information is currently available on the effect of individual AA on GLP-1 in pigs.

Based on performance data, it has been shown that excess dietary levels of Lys, Met, Thr, and Trp reduce feed intake and growth in pigs (Edmonds et al., 1987). In addition, the impact of Lys on feed intake was associated with an increased CCK expression in weaner pigs (Yin et al., 2017). A potential mediation of gut peptides in the appetite modulation by Met, Thr, and Trp seems to remain unknown.

Overall, there is limited literature on the secretion of gut hormones and their relevance to appetite regulation in pigs. The effect of AA on CCK and GLP-1 is a subject that merits further investigation. Studying the individual effect of AA on CCK and GLP-1 secretion may help determine the anorexigenic potential of dietary AA. We hypothesized that the most limiting (Lys and Met), aromatic (Trp and Phe), and branched-chain (**BCAA**; Leu and Ile) AA will significantly stimulate CCK and/or GLP-1 secretion in pigs. The main objective of this study was to evaluate the effect of physiologically relevant doses of the 20 proteinogenic AA on the intestinal release of CCK and GLP-1 in young pigs.

## Materials and Methods

### Animal ethics

All experimental treatments and procedures were approved by The University of Queensland Animal Ethics Committee (Animal Ethics Certificate: CNFS/568/16).

### Chemicals

The 20 proteinogenic AA: Ala, Arg, Asn, Asp, Cys, Gln, Glu, Gly, His, Ile, Leu, Lys, Met, Phe, Pro, Ser, Thr, Trp, Tyr, and Val (reagent grade; purity ≥97%), were purchased from Sigma-Aldrich (Castle Hill, New South Wales, Australia). Triton-X 100, cytotoxicity detection kit (PLUS) LDH (Roche Diagnostics), and the chemicals for the preparation of the Krebs–Ringer bicarbonate (**KRB**)/HEPES buffer were purchased from Sigma-Aldrich. The KRB buffer containing magnesium chloride (0.0468 g/L), potassium chloride (0.34 g/L), sodium chloride (7.0 g/L), sodium phosphate dibasic (0.1 g/L), sodium phosphate monobasic (0.18 g/L), d-glucose (1.8 g/L), and HEPES (5,579 g/L) was adjusted to pH 7.4 before use.

### Animals, housing, and feeding

Six 25-d-old male piglets (Domestic Landrace × Large White; body weight = 6.94 ± 0.29 kg) were sourced from the experimental farm of SunPork Solutions Ltd (SunPork Pty Ltd, Eagle Farm, Queensland, Australia) located in Westbrook (Queensland, Australia). The pigs were housed in an environmentally controlled room with fully slatted floor pens at the Herston Medical Research Centre of The University of Queensland (Herston Campus, Queensland, Australia). The temperature in the nursery was thermostatically set between 27 and 28 °C. Pigs had ad libitum access to water and feed for the duration of the experiment. The starter diet was formulated to meet or exceed all essential amino acids (**EAA**) requirements (Lys, Met, Thr, and Trp) without the need for synthetic AA supplementation (Table 1). Following a 7-d adaptation period, piglets were humanely killed (Lethabarb; 162.5 mg/kg) to acquire intestinal samples from the duodenum, jejunum, and ileum.

**Table 1.** Composition of the experimental diet (as fed basis)

	%
Ingredients, %	
Wheat	60.80
Soya bean full	16.00
Blood meal	3.00
Meat meal	6.55
Fish meal	4.25
Chocolate milk powder	5.00
Single cell protein	2.50
Vegetable oil	1.50
Salt	0.15
Choline chloride 60%	0.04
Vitamin and mineral premix^1^	0.20
Calculated nutrient content, %	
Crude protein	24.97
Digestible energy, MJ/kg	15.25
Calcium	1.18
Phosphorus	0.86
Lysine	1.40
Methionine	0.44
Threonine	0.94
Tryptophan	0.28
Met/Lys	0.31
(Met + Cys)/Lys	0.60
Trp/Lys	0.20
Thr/Lys	0.67
Analyzed composition^2^, %	
Crude protein	25.13
Moisture	8.19
Ash	5.31
Crude fiber	2.69
Ether extract	6.90
Lysine	1.33
Methionine	0.42
Threonine	0.95
Tryptophan	0.30
Glycine	1.34
Histidine	0.74
Arginine	1.53
Alanine	1.45
Tyrosine	0.70
Valine	1.22
Serine	1.14
Phenylalanine	1.28
Isoleucine	0.88
Leucine	1.88
Glutamic acid	4.47
Proline	1.69
Hydroxyproline	0.27
Aspartic acid	2.10

Premix composition (as fed basis): vitamin A, 10,000 IU/kg; vitamin D3, 1,800 IU/kg; vitamin E, 100 mg/kg; vitamin K3, 5 mg/kg; vitamin B1, 3 mg/kg; vitamin B2, 6 mg/kg; niacin, 30 mg/kg; pantothenic acid, 30 mg/kg; pyridoxine, 4 mg/kg; biotin, 0.3 mg/kg; folic acid, 2.5 mg/kg; vitamin B12, 0.04 mg/kg; iron, 100 mg/kg; iodine, 0.7 mg/kg; manganese, 45 mg/kg; selenium, 0.3 mg/kg; zinc, 120 mg/kg; cobalt, 0.3 mg/kg; copper, 10 mg/kg.

Based on laboratory proximal and AA analysis.

### Intestinal tissue collection

On day 8 of the trial, animals were humanely killed. The small intestine was removed from the pylorus to the ileocecal valve and the length was measured. Intestinal segments were quickly (within 10 min) collected from the duodenum (5 cm distal from the pylorus), proximal jejunum (approx. 100 to 120 cm distal from the pylorus; based on the total length of the small intestine), and ileum (5 cm proximal from the ileocecal valve). The sampling locations were selected based on previous data published by Adeola and King (2006). Intestinal segments were stored in ice-cold KRB/HEPES buffer bubbled with O_2_/CO_2_ (95%/5%), to prevent ischemia, and transported within 20 min (one pig at a time) to Lab C213 at Hartley Teakle building 83 at The University of Queensland (St Lucia Campus, Queensland, Australia) for further processing.

### Primary intestinal cell culture

Intestinal segments were used in a porcine primary cell culture adapting the method published by Voortman et al. (2012). In short, tissue segments were cut open longitudinally, cleaned from debris using buffered KRB/HEPES, and stripped of their outer muscle layer before equally sized circular samples (approximately 1.13 cm^2^, 68 samples per intestinal segment) were excised using a 12 mm biopsy punch (Acuderm Inc., Fort Lauderdale, Florida, USA). The circular tissue samples were transferred into 24-well plates (Thermofisher Scientific, Waltham, Massachusetts, USA) filled with 500 μL ice-cold KRB/HEPES buffer (pH 7.4) and kept at room temperature for 30 min before being placed in a humidified incubator at 37 °C and 5% v/v CO_2_. Following a preincubation of 1 h, the media within each well was replaced with a pre-warmed KRB/HEPES buffer (37 °C, 500 μL with pH 7.4) containing no glucose but 1 of the 20 AA or a control (buffer with no added AA or glucose) and incubated for an additional 1 h at 37 °C and 5% v/v CO_2_. Triplicates of untreated or control samples were used for the evaluation of the gut hormone secretion and gene expression levels across the small intestine segments of piglets. All AA were tested at a concentration of 10 mM (except for Tyr which was tested at 2.5 mM due to low solubility) based on previously published data on AA intestinal concentrations following protein digestion in pigs (Cho and Bayley, 1972). After incubation, the media from each well was collected into Eppendorf tubes and stored at −80 °C for future hormone analysis. Likewise, following incubation, tissue samples were transferred into Eppendorf tubes filled with RNAlater and left at room temperature for 24 h before being placed at −80 °C for future mRNA analysis. To check tissue viability, lactate dehydrogenase (**LDH**) activity (a cytosolic enzyme released following cell abrasion) was measured in samples and compared with positive controls (tissue samples treated with 1% of Triton-X 100) following the (PLUS) LDH kit’s instructions. The collected media for the evaluation of LDH levels was stored at 4 °C until analysis (within 24 h of collection).

### Gut hormones and LDH secretion analysis

Concentrations of CCK released by EEC were analyzed using a Porcine Cholecystokinin ELISA kit (MBS264395) from MyBioSource (San Diego, California, USA). The inter-assay coefficient of variance (**CV**) for the CCK kit was 7.5% and the intra-assay CV was 3.2%. GLP-1 levels were analyzed using the Glucagon-Like Peptide-1 (Total) ELISA kit (EZGLPT1-36K) from Merck Millipore (Burlington, Massachusetts, USA). Inter-assay CV was 7.8%, whereas the intra-assay CV was 2.1%. When necessary, samples were diluted in assay buffer to obtain values within the detection range of the kit according to the manufacturer’s guidelines. The optical density of the ELISA plate wells was measured in a BMG FLUOstar OPTIMA Microplate Reader (BMG Labtech, Mornington, Victoria, Australia). To check the viability of the intestinal tissues incubated, LDH activity was determined using a Roche LDH reagent kit PLUS (Sigma-Aldrich).

### CCK and proglucagon (GCG) RNA extraction and RT-qPCR analysis

GLP-1 gene expression in the form of its protein precursor GCG, also expressed in L-cells, was determined in this study (Baggio and Drucker, 2007). RNA extraction was performed following the standard procedure recommended by the manufacturer. In brief, the initial extraction of RNA from the intestinal mucosa samples was performed using Trizol Reagent (Cat. No. 15596026; Invitrogen, Carlsbad, California, USA). The PureLink RNA Mini Kit (Cat. No. 12183018A; Invitrogen), was used for the isolation of high-quality total RNA. RNA quality and concentration in tissue samples were measured using an Invitrogen Nano Drop spectrophotometer (NanoDrop 8000, Thermofisher Scientific). Next, the cDNA synthesis was performed with QuantiTect Reverse Transcription Kit (Cat. No. 205313; Qiagen, Hilden, Germany). Primers for CCK (forward: 5ʹ-CAGGCTCGAAAAGCACCTTC-3ʹ, reverse: 5ʹ-GCGGGGTCTTCTAGGAGGTA-3ʹ, 157 bp), GCG (forward: 5ʹ-AGAACTCCGCCGCAGACA-3ʹ, reverse: 5ʹ-TAAAGTCTCGGGTGGCAAGATT-3ʹ, 65 bp), and GAPDH (forward: 5ʹ-TGGTGAAGGTCGGAGTGAAC-3ʹ, reverse: 5ʹ-GAAGGGGTCATTGATGGCGA-3ʹ, 104 bp) used in this study have previously been published by Tian et al. (2019; for CCK and GCG) or da Silva et al. (2014; for GAPDH). The reaction volume (10.05 uL) for the real-time PCR contained the following: 5 µL of SYBR Green master mix solution, 3 µL of RNAs free water, 1 µL of cDNA sample, 0.5 µL forward and reverse PCR primers, and 0.05 µL of ROX reference dye solution. The RT-qPCR analysis followed a modified version of the program published by Van Liefferinge et al. (2021). In brief, the PCR program was set for denaturation at 95 °C for 2 min, followed by 40 cycles of 95 °C for 15 s and 60 °C for 60 s using QuantStudio 6, Thermofisher Scientific. All samples were measured in triplicate. GAPDH was used as a reference gene for the relative calculations of gene expression levels following the Pfaffl method (Pfaffl, 2001).

### Statistical analysis

Statistical analysis was performed using R software (RStudio, Inc., Boston, Massachusetts, USA). Gut hormone secretion and gene expression data across intestinal segments were analyzed using a two-way ANOVA considering “tissue” as a fixed effect and “pig” as a random effect, and their interaction, followed by a Tukey post hoc test. To compare the effect of individual AA on CCK and GLP-1 secretion with control-treated samples, a two-way ANOVA was run including “AA” as a fixed factor and “pig” as a random factor, and their interaction. Within each pig, each of the 20 individual AA were independently tested on 3 tissue samples (biological replicates) from the intestinal segment identified with the highest secretion and expression level of CCK or GLP-1. Data are presented as the mean ± SEM of absolute amounts or percentage of control. Results were considered statistically significant when the *P* < 0.05. Values of 0.05 < *P* < 0.1, were noted as trends. The number of samples (*n*) refers to the number of pigs used.

## Results

LDH levels were measured in the duodenum, jejunum, and ileum samples to determine the integrity of the primary intestinal cultures. LDH leakage from samples was 4.45 ± 2.12%, 8.60 ± 3.13%, and 6.56 ± 1.56% for the duodenum, jejunum, and ileum samples, respectively. Intestinal samples for gut hormone analysis must contain ideally an LDH leakage of 10% or less relative to the positive controls (tissue samples treated with Triton-X 100) indicating that the results validated the integrity of the samples (Voortman et al., 2012; Ripken et al., 2014).

**Figure 1.**
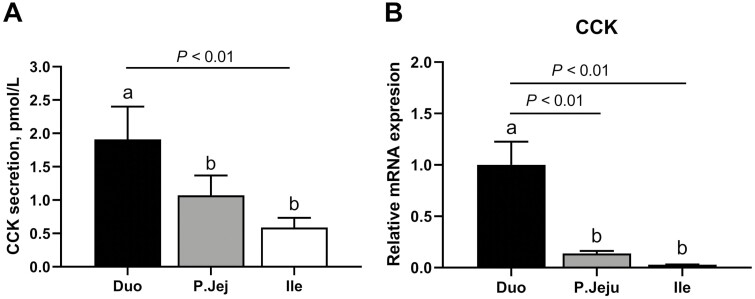
Cholecystokinin (CCK) secretion and gene expression levels along the small intestine of weaner pigs. Tissue hormone levels (measured in supernatant) (A) and mRNA expression (B) of CCK in primary cultures of duodenum (Duo), proximal jejunum (P. Jeju), and ileum (Ile) of postweaning pigs incubated for 1 h in KRB/HEPES free glucose buffer (*n* = 6). Data are expressed as the mean + SEM. Different letters (a, b, and c) indicate significant differences (*P* < 0.05).

**Figure 2.**
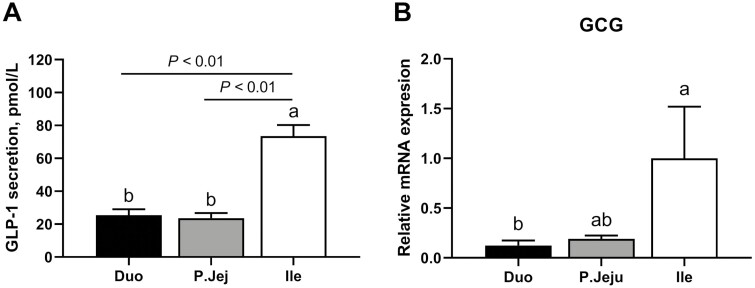
Glucagon-like-peptide 1 (GLP-1) secretion and proglucagon (GCG) gene expression levels along the small intestine of weaner pigs. Tissue hormone levels of GLP-1 (measured in supernatant) (A) and GCG gene expression (B) in primary cultures of duodenum (Duo), proximal jejunum (P. Jeju), and ileum (Ile) of postweaning pigs incubated for 1 h in KRB/HEPES free glucose buffer (*n* = 6). Data are expressed as the mean + SEM. Different letters (a and b) within the same graph indicate significant differences between tissues (*P* < 0.05).

The results of the concentration and tissue expression of CCK and GLP-1 in the duodenum, jejunum, and ileum samples incubated for 1 h in KRB/HEPES buffer with no added glucose are shown in Figures 1 and 2, respectively. CCK released from duodenum was significantly higher than from the proximal jejunum (*P* < 0.05) or ileum (*P* < 0.01) reaching 1.91 ± 1.1, 1.07 ± 0.67, and 0.59 ± 0.32 pmol/L, respectively. Similarly, the gene expression data showed higher levels of the CCK mRNA in the duodenum than in the proximal jejunum (*P* < 0.05) or ileum (*P* < 0.01; Figure 1). In contrast, GLP-1 concentrations were significantly (*P* < 0.01) higher in the ileum than in the jejunum or duodenum cultures reaching 73.58 ± 14.84, 23.61 ± 6.95, and 25.46 ± 7.96 pmol/L, respectively. Consistently, an expression pattern of GCG was also identified predominantly in the ileum (Figure 2). A Tukey test revealed significant differences in the GCG mRNA abundance between ileum and duodenum (*P* < 0.05). Based on these results, the duodenum and ileum were selected for the testing of the effect of individual AA on CCK or GLP-1, respectively.

**Figure 3.**
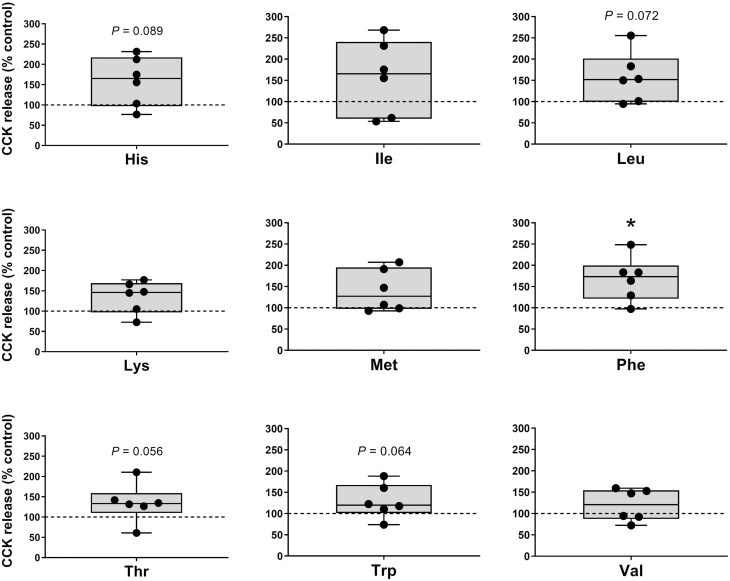
Duodenal cholecystokinin (CCK) secretion following essential amino acid (EAA) stimulation in weaner pigs. CCK secretion form porcine duodenum cultures following a 1 h incubation with EAA (His, Ile, Leu, Lys, Met, Phe, Thr, Trp, and Val) at 10 mM. Data are expressed as percentage of control (KRB buffer alone = dotted line). *n* = 6, each data point within boxplots represents the average of three biological replicates within each pig. *P < 0.05.

**Figure 4.**
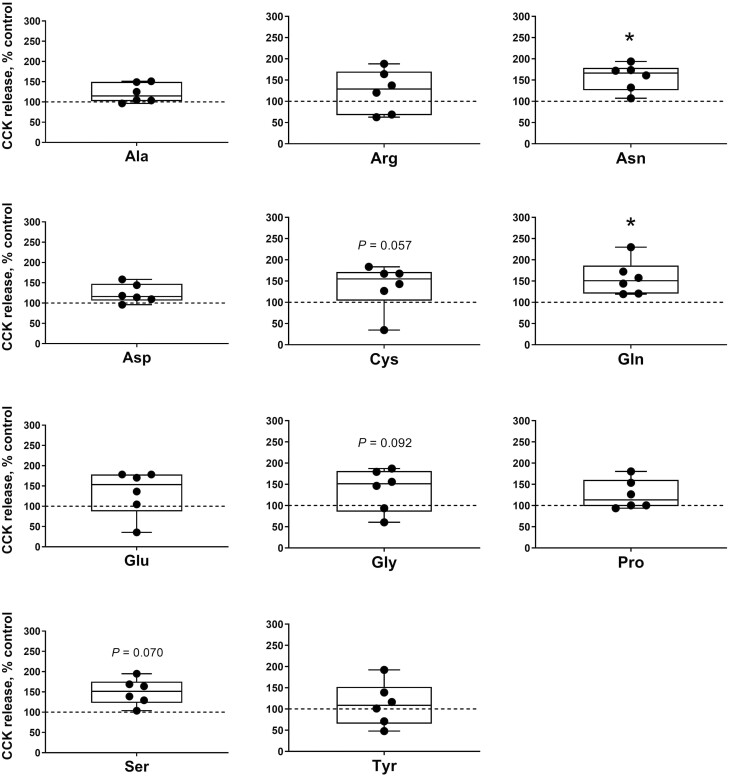
Duodenal cholecystokinin (CCK) secretion following non-essential amino acid (NEAA) stimulation in weaner pigs. CCK secretion form porcine duodenum cultures following a 1 h incubation with NEAA at 10 (Ala, Arg, Asn, Asp, Cys, Gln, Glu, Gly, Pro, and Ser) or 2.5 mM (Tyr). Data are expressed as percentage of control (KRB buffer alone = dotted line). *n* = 6, each data point within boxplots represents the average of three biological replicates within each pig. *P < 0.05.

The effects of incubating duodenum samples with each individual 9 EAA or 11 non-essential amino acids (**NEAA**) in pigs on CCK secretion are illustrated in Figures 3 and 4, respectively. Regarding EAA, the incubation with Phe (2.64 ± 0.33 pmol/L) significantly (*P* < 0.05) increased whereas with Leu, His, Trp, and Thr (2.46 ± 0.38, 2.50 ± 0.39, 2.03 ± 0.26, and 2.11 ± 0.31 pmol/L, respectively) tended (*P* < 0.1) to increase CCK release in duodenum compared with the control samples (1.57 ± 0.23 pmol/L). Among the NEAA, Asn and Gln (2.49 ± 0.20 and 2.50 ± 0.27 pmol/L, respectively) significantly increased (*P* < 0.05) whereas Gly, Ser, and Cys (2.18 ± 0.32, 2.38 ± 0.21, and 2.18 ± 0.35 pmol/L, respectively) resulted in a trend (*P* < 0.1) to stimulate CCK secretion from the duodenum when compared with the KRB/HEPES free glucose buffer control samples.

**Figure 5.**
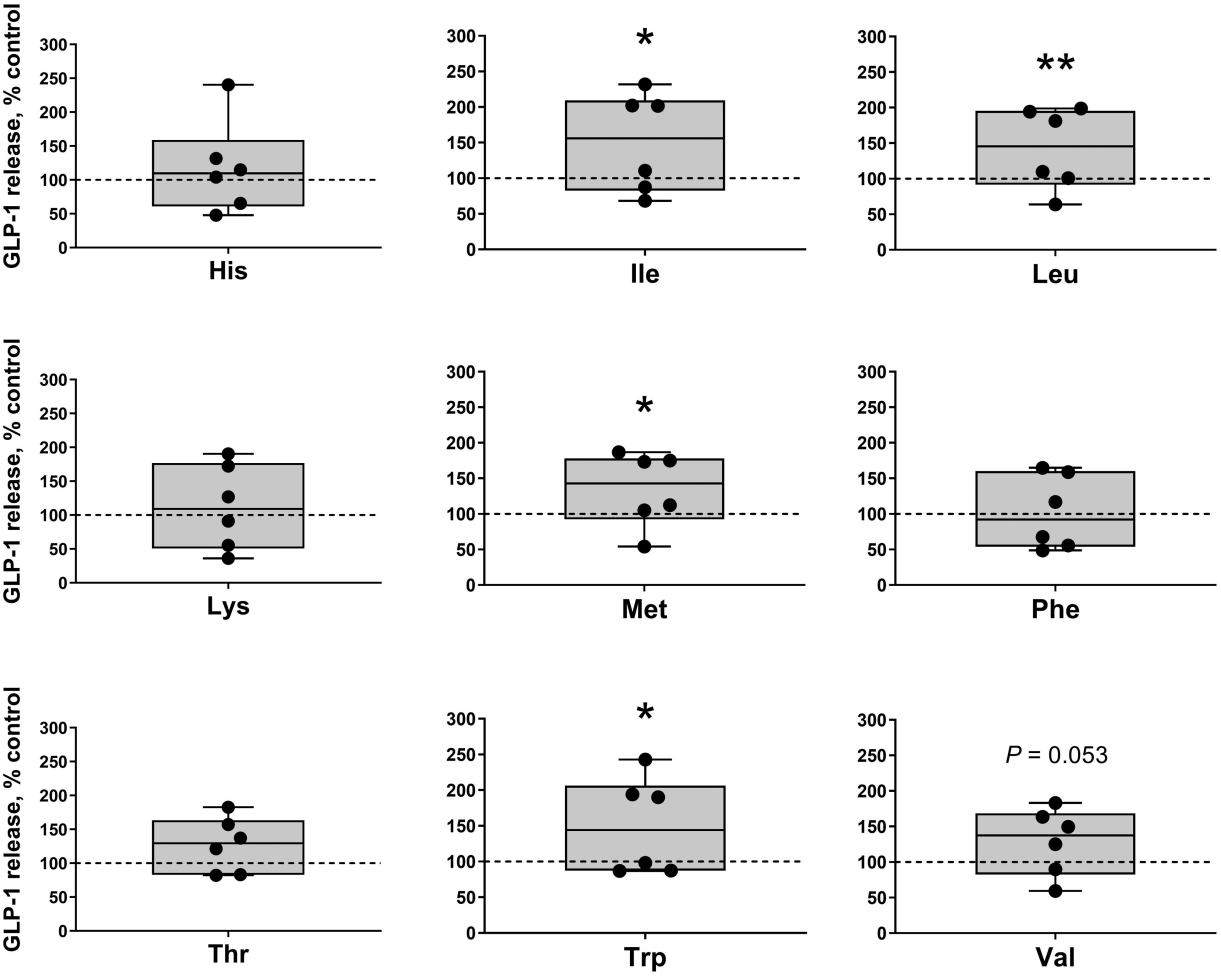
Ileal glucagon-like peptide 1 (GLP-1) secretion following essential amino acid (EAA) stimulation in weaner pigs. GLP-1 secretion from porcine ileum cultures following a 1 h incubation with EAA (His, Ile, Leu, Lys, Met, Phe, Thr, Trp, and Val) at 10 mM. Data are expressed as percentage of control (KRB buffer alone = dotted line). *n* = 6, each data point within boxplots represents the average of three biological replicates within each pig. *P < 0.05, **P < 0.01.

**Figure 6.**
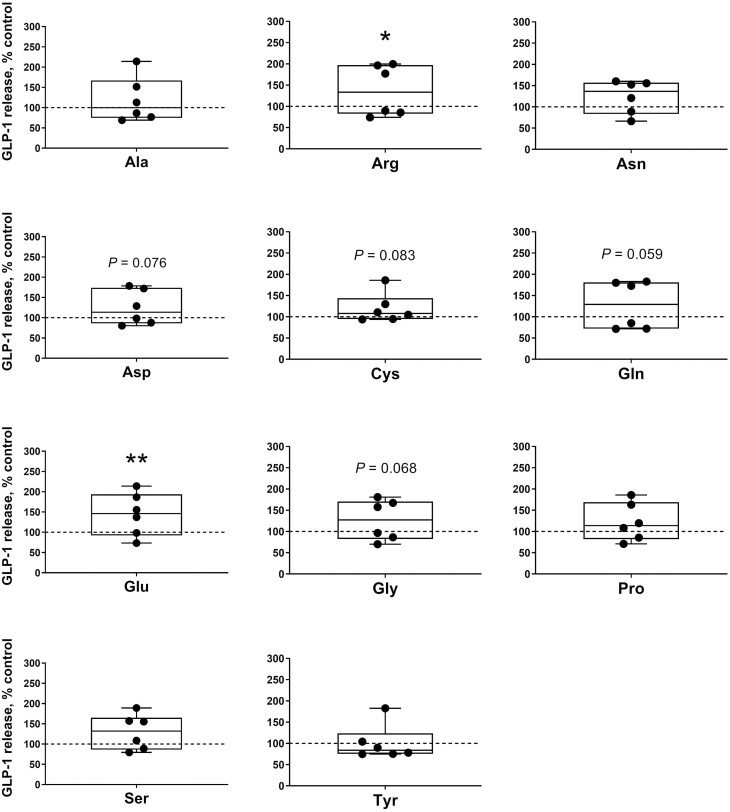
Ileal glucagon-like peptide 1 (GLP-1) secretion following non-essential amino acid (NEAA) stimulation in weaner pigs. GLP-1 secretion from porcine ileum cultures following a 1 h incubation with NEAA at 10 (Ala, Arg, Asn, Asp, Cys, Gln, Glu, Gly, Pro, and Ser) or 2.5 mM (Tyr). Data are expressed as percentage of control (KRB buffer alone = dotted line). *n* = 6, each data point within boxplots represents the average of three biological replicates within each pig. *P < 0.05, **P < 0.01.

The effects of incubating each single EAA or NEAA on the secretion of GLP-1 from the ileum samples are shown in  Figures 5 and 6. From the EAA, Ile, Leu, Met, and Trp (305.93 ± 57.79, 288.13 ± 47.56, 273.73 ± 43.52, and 305.29 ± 56.07 pmol/L, respectively) significantly triggered GLP-1 release (*P* < 0.05) whereas Val (261.53 ± 38.91 pmol/L) showed a tendency (*P* < 0.1) to increase the gut peptide secretion compared with the control (201.16 ± 42.57 pmol/L). Regarding NEAA, Arg and Glu (279.06 ± 49.74 and 293.31 ± 43.88 pmol/L, respectively) significantly increased (*P* < 0.05) whereas Asp, Cys, Gln, Gly, and Ser (253.15 ± 35.61, 244.22 ± 28.91, 259.49 ± 46.86, 257.50 ± 39.43, and 264.07 ± 36.37 pmol/L, respectively) tended (*P* < 0.1) to trigger GLP-1 released from ileum when compared with the saline controls.

## Discussion

An ex vivo model sampling the three main sections of the porcine small intestine (duodenum, jejunum, and ileum) was selected to characterize the effect of free AA on the two main anorexigenic gut peptides known in pigs CCK and GLP-1. The results showed a pattern of secretion and gene expression of CCK (in the absence of AA) characteristic of the porcine small intestine with a higher abundance in the duodenum than in the jejunum or ileum. These results aligned with those previously described in growing and finishing pigs (Bacarese-Hamilton et al., 1984; Voortman et al., 2012). In contrast, GLP-1 secretion levels were highest in the ileum compared with the other segments of the small intestine studied. This secretion pattern was confirmed with the mRNA expression levels of GCG. These results are also consistent with the current literature (Voortman et al., 2012; Wewer Albrechtsen et al., 2016). Taken together, these secretion patterns validate the ex vivo model used in this research project.

The experimental approach was novel in that, to the best of our knowledge, never have all the 20 individual proteinogenic AA had been tested for CCK and GLP-1 in pigs. The results showed several novel observations such as that none of the AA studied significantly stimulated the release of both gut peptides from EEC. This finding fits well with previous reports where AA sensors and receptors and gut hormones are not homogeneously expressed along with the pig GIT, rather they follow specific patterns. For example, our results mimic the observations of van der Wielen et al. (2014) who reported high levels of expression of CCK in the porcine duodenum and in scattered locations along the jejunum but not in the ileum. In addition, the glucagon-like peptide 1 receptor (**GLP-1R**) was co-expressed in similar areas to CCK in the duodenum. In a recent review, Müller et al. (2021) were able to map the sites of expression of the main AA sensors and gut peptides in the porcine GIT. These differences in the pattern of expression of AA sensors may explain the singularities of how different dietary AA stimulate the secretion of CCK or GLP-1 (Tian et al., 2019).

In addition, the results showed that none of the four most limiting EAA in pig diets (Lys, Met, Thr, and Trp), triggered the release of CCK in the duodenum. This is potentially relevant to commercial formulation practices where balanced diets are often supplemented with these limiting EAA using crystal and synthetic forms. In previous work, pigs were shown to be highly tolerant to dietary excesses of non-bound EAA supplements (Edmonds and Baker, 1987; Edmonds et al., 1987). Free EAA in the diet are thought to be absorbed early in the proximal segments of the small intestine before enzymatic digestion occurs (Yen et al., 2004). Thus, from an evolutionary point of view, most AA sensors would be anticipated further down the GIT in synchrony with enzymatic digestion (Silk et al., 1985; Bröer, 2008). Consistent with this principle, several of the EAA triggered a significant response from the ileum primary culture by means of increasing the release of GLP-1. These were the three BCAA (Leu, Ile, and Val—it is noted that the latter was a trend where *P* = 0.053-), and Met and Trp.

The results on BCAA-induced GLP-1 secretion align with those previously described in human and mice using intestinal ex vivo and in vitro models (Reimann et al., 2004; Chen and Reimer, 2009). In addition, these results agree with previous reports describing a satiating effect of dietary BCAA in pigs (Wiltafsky et al., 2010; Wessels et al., 2016; Kwon et al., 2019). Leu consumption has also been associated with increased insulin blood levels in piglets, a process known to be mediated by GLP-1 or other incretins such as the gastric inhibitory polypeptide (Zhang et al., 2019). Met and Trp also triggered the release of GLP-1 in the porcine ileum. The effect of Trp on GLP-1 secretion is consistent with previous reports in rodents (Mace et al., 2012). In pigs, dietary excesses of Trp (4%) and Met (2%) have been related to reduce feed intake when compared with a standard weaner diet (Edmonds and Baker, 1987). Hence, our data suggest that Met and Trp may exert an anorexigenic effect associated with their capacity to stimulate GLP-1 secretion from the ileum. In addition, Trp has been shown to increase ghrelin secretion and feed intake in pigs (Zhang et al., 2007). While this study did not involve ghrelin, the significant impact on GLP-1 suggests that Trp may play a relevant role in orchestrating hunger-satiety in the gut consisting of a sequence of orexigenic (ghrelin) followed by anorexigenic stimulus (GLP-1) in a sequential fashion as food progresses from stomach to the distal small intestine, respectively.

The lack of effect of EAA Leu, Ile, and Trp on the release of CCK in pigs were in contrast with some recent publications (Zhao et al., 2018; Tian et al., 2019). This could be related to the limitations of the ex vivo model used in this study which disallowed feedback mechanisms from other GIT segments (Cummings and Overduin, 2007; Furness et al., 2013). On the one hand, it seems unlikely that EAA may directly stimulate sensors in the duodenum (before enzymatic digestion). On the other hand, the co-expression of CCK and the GLP-1R shown by van der Wielen et al. (2014) is consistent with a feedback mechanism supported by our data where these EAA would trigger GLP-1 secretion from the ileal epithelia which in turn would retro-activate the GLP-1R in the duodenum (but not in an ex vivo model). In addition, differences in methodology such as AA concentration, incubation time, culture media or buffer (absence of divalent cations, e.g., Ca2+), and intestinal segments tested, among other factors, could have contributed as well to the lower CCK responses observed in this compared with previous experiments.

Phe was the only EAA, which significantly stimulated CCK release directly from the primary culture of the porcine duodenum. These results are consistent with previous intestinal ex vivo and in vitro studies on Phe-induced CCK release in rodents and pigs (Mangel et al., 1995; Wang et al., 2011; Feng et al., 2019). While the nutritional relevance of Phe is unclear in pigs, the current results advocate for further investigations to describe its role in modulating appetite.

Several NEAA have been identified as major energy substrates of the intestinal mucosa and constituents of proteins relevant to the gut barrier function (Wu, 2013). Most notably Gln, Asn, Glu, and Arg have been associated with gut health in pigs (Chalvon-Demersay et al., 2021). In our studies, the two amide AA (Gln and Asn) were significantly involved in releasing CCK from the duodenum, while Glu and Arg increased the release of GLP-1 from the ileum. Consistent with the results, amide AA have previously been shown to delay gastric emptying, a physiological response associated with CCK release (Moran and McHugh, 1982; Nishi et al., 2003). The amide group can be easily hydrolyzed; thus, these two AA play a crucial role in nitrogen (N) exchange via ammonia (NH_3_) transport between tissues. However, N is an essential nutrient for the development of the microbiota as well. Limiting the availability of N for bacterial growth is fundamental to control pathogen infection. While admittedly speculative, the high responsiveness of the proximal GIT to amide AA by slowing down the rate of passage may respond to the need of limiting the N available for microbial use. Similarly, a significant impact of Glu and Arg inducing GLP-1 release aligns with previous results in rodents (Reimann et al., 2004; Mace et al., 2012). In pigs, high supplementation of Glu (as monosodium glutamate) has been related to delayed gastric emptying, lower feed intake, and growth (Bauchart-Thevret et al., 2013; Li et al., 2018). Glu-induced GLP-1 release may be related to its use as a main metabolic fuel by the gut epithelium (Burrin and Stoll, 2009). Given the affinity of Glu for the T1R1-T1R3 in pigs, the potential involvement of the receptor in Glu-induced GLP-1 release merits further investigation (Roura et al., 2011).

These data support the notion that gut hormone release is dependent on the nature and characteristics of the AA (potentially related to their side-chain property). Additional studies are required to confirm these initial observations.

## Conclusions

This study consisted of systematic testing of all 20 proteinogenic AA on the release of anorexigenic gut peptides CCK and GLP-1. Whereas the duodenum is the small intestinal segment with the highest CCK gene expression and secretion, GLP-1/GCG is mainly secreted in the ileum of weaned pigs. Collectively, the data presented in this manuscript provide evidence that both EAA and NEAA regulate key metabolic processes associated with appetite control in pigs. In particular, gut responses to EAA seem to be stronger in post enzymatic processes such as the release of GLP-1 from EEC in the ileum. In contrast, proximal responses to NEAA (particularly amide AA) are robust leading to an early release of CCK in the duodenum.

## Abbreviations

AA: amino acids
CaSR: calcium-sensing receptor
CCK: cholecystokinin
EAA: essential amino acids
EEC: enteroendocrine cells
GCG: proglucagon
GIT: gastrointestinal tract
GLP-1: glucagon-like peptide 1
GLP-1R: glucagon-like peptide 1 receptor
KRB: Krebs–Ringer bicarbonate
LDH: lactate dehydrogenase
NEAA: nonessential amino acids
T1R1-T1R3: umami taste receptor
